# Author Correction: 2600-years of stratospheric volcanism through sulfate isotopes

**DOI:** 10.1038/s41467-019-10539-9

**Published:** 2019-06-17

**Authors:** E. Gautier, J. Savarino, J. Hoek, J. Erbland, N. Caillon, S. Hattori, N. Yoshida, E. Albalat, F. Albarede, J. Farquhar

**Affiliations:** 1 Univ. Grenoble Alpes, CNRS, IRD, Grenoble INP, Institut des Géosciences de l’Environnement (IGE), 54 rue Molière, 38058 Grenoble Cedex 9, France; 2 Department of Geology and Earth System Science Interdisciplinary Center (ESSIC), University of Maryland, College Park, MD 20742 USA; 3 Department of Chemical Science and Engineering, School of Materials and Chemical Technology, Tokyo Institute of Technology, G1-17, 4259 Nagatsuta-cho, Midori-ku, Yokohama, Kanagawa, 226-8502 Japan; 4 Earth-Life Science Institute, Tokyo Institute of Technology, 2-12-1-IE-1 Ookayama, Meguro-ku, Tokyo, 152-8550 Japan; 5 Ecole Normale Supérieure de Lyon, CNRS and University of Lyon, 9 rue du Vercors, 69364 Lyon Cedex 7, France

**Keywords:** Cryospheric science, Cryospheric science

Correction to: *Nature Communications* 10.1038/s41467-019-08357-0, Published online 28 Jan 2019.

The authors became aware of a mistake in the data and axis labeling in Fig. 2 in the original version of the Article. Specifically, the authors mistakenly copied and pasted a formula for background correction instead of the actual values.

As a result of this, Fig. 3 was updated to replace the incorrect label ‘sulfate flux (kg km^−2^)’ with the correct ‘sulfate concentrations (ng g^−1^)’ on the far-left *y*-axes in both panels, and to add the correct data for Δ^33^S, as given by the red dotted lines. The correct version of Fig. 3 is shown below as Fig. 1, which replaced the previous incorrect version, shown below as Fig. 2.

**Figure 1:**
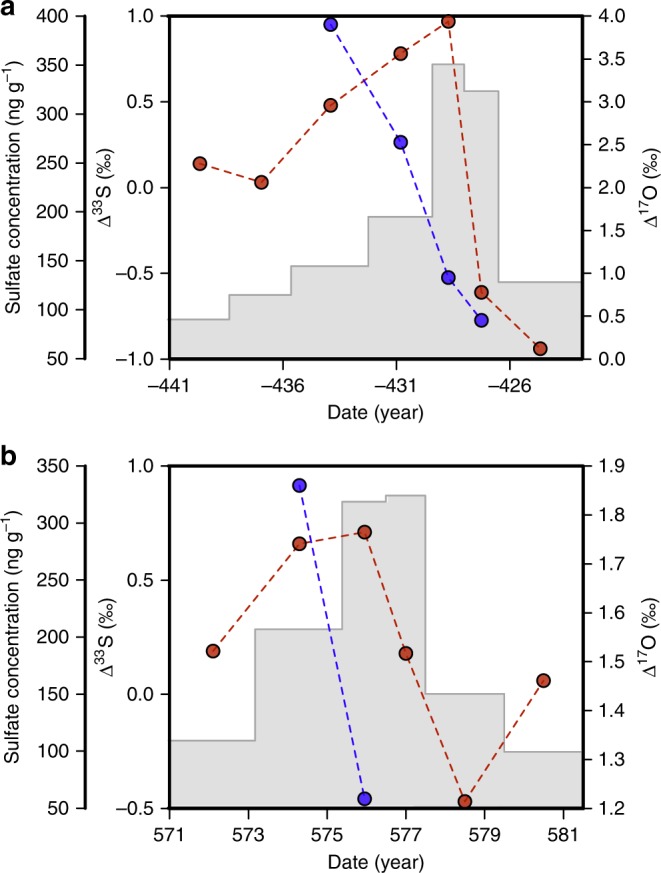
Fig. 1

**Figure 2:**
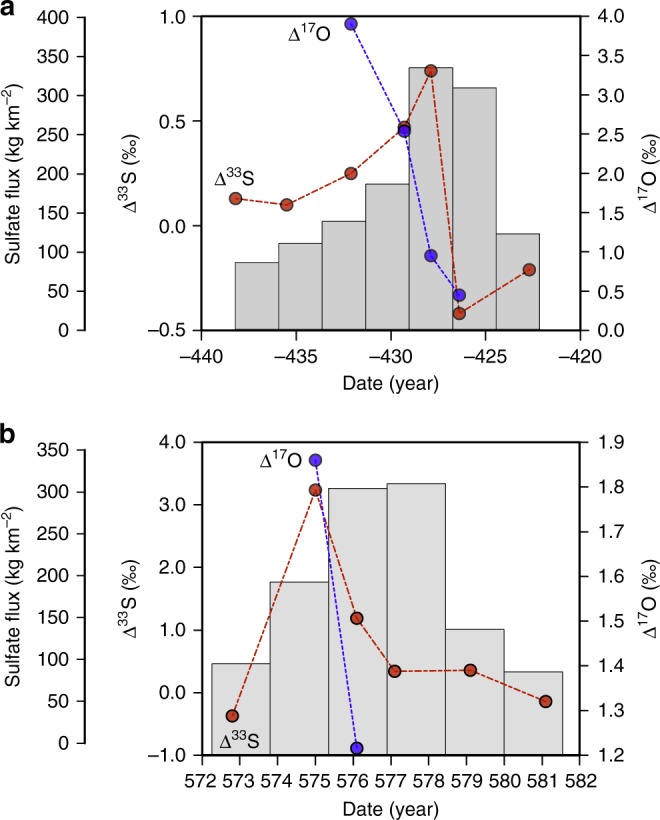
Fig. 2

This has been corrected in both the PDF and the HTML versions of the Article. The findings and interpretations in the original Article are based on the correct dataset, and this error does not affect the original discussion or conclusions of the Article. The authors apologize for the confusion caused by this mistake.

