# Supplementary material for: 2600-years of stratospheric volcanism through sulfate isotopes
Source: Nat Commun. 2019 Jan 28;10:466. doi: 10.1038/s41467-019-08357-0 (PMC6349899; doi:10.1038/s41467-019-08357-0)
Supplement: Supplementary file 1 — Supplementary Information [file 41467_2019_8357_MOESM1_ESM.pdf]

Supplementary material for

2600-years of stratospheric volcanism through sulfate isotopes

Gautier *et al.*

Event	Date CE	Flux (kg.km-2)	$\delta^{34}S$ (‰)	*1 σ ($\delta^{34}S$)*	$\Delta^{33}S$ (‰)	*1 σ ($\Delta^{33}S$)*	Source
**1 Pinatubo**	1991	10	21,42	*0,25*	1,19	*0,12*	**S**
**2 Agung**	1963	6	15,93	*0,25*	1,41	*0,12*	**S**
**3,1**	1890	1	14.26 (R)	*0,60*	0.04 (R)	*0,02*	**T**
**3.2 Krakatau**	1884	9	15.02 (R)	*0,6*	0.2 (R)	*0,02*	**S**
**4**	1861	2	17.45 (R)	*0,6*	0.27	*0,19*	**S**
**5 Cosigüina**	1835	10	8,99	*2,7*	0,43	*0,1*	**S**
**6.1 Tambora**	1816	13	-3,88	*3,99*	-0,63	*0,14*	**S**
**6.2 UE 1809**	1810	18	2,27	*1,53*	0,19	*0,05*	**S**
**7**	1762	5	20,63	*4,6*	0,75	*0,17*	**S**
**8**	1694	11	5,64	*1,38*	0,39	*0,05*	**S**
**9**	1672	7	11,57	*2,35*	0,62	*0,08*	**S**
**10**	1642	16	14,53	*1,2*	1,11	*0,04*	**S**
**11**	1623	7	13.37 (R)	*0,6*	0.03 (R)	*0,02*	**T**
**12.1 Huainaputina**	1601	9	6,77	*2,61*	0,94	*0,09*	**S**
**12.2 Ruang**	1597	6	23,21	*4,6*	2,29	*0,17*	**S**
**13 Kuwae**	1458	42	8,2	*1,43*	0,73	*0,05*	**S**
**14**	1374	7	13,58	*3,59*	0,68	*0,13*	**S***
**15**	1346	15	15,99	*1,83*	1,51	*0,06*	**S**
**16.1**	1285	30	15,39	*4,44*	0,84	*0,16*	**S**
**16.2**	1276	34	5,5	*0,68*	0,37	*0,02*	**S**
**16.3**	1270	10	11.98 (R)	*0,6*	0.08 (R)	*0,02*	**T**
**16.4 Samalas**	1259	85	23,74	*0,93*	2,22	*0,03*	**S**
**17**	1230	30	18,15	*2,26*	1,38	*0,08*	**S**
**18.1**	1193	45	14,44	*0,81*	0,78	*0,03*	**S**
**18.2**	1172	22	12,79	*1,83*	0,22	*0,06*	**S**
**19**	1110	14	0,25	*4,14*	-0,96	*0,15*	**S**
**20**	1073	9	13.47 (R)	*0,6*	0.01 (R)	*0,02*	**T**
**21.1**	1040	5	15.73 (R)	*0,6*	0.36 (R)	*0,02*	**S***
**21.2**	1031	5	23,3	*4,94*	0,76	*0,18*	**S**
**22**	960	13	11,75	*1,83*	0,6	*0,06*	**S***
**23**	901	7	19,34	*2,35*	1,48	*0,08*	**S**
**24.1**	857	6	3,8	*5,69*	-0,79	*0,21*	**S**
**24.2**	852	6	3.53 (R)	*0,6*	0.25 (R)	*0,02*	**S**
**25**	820	9	16,47	*4,14*	0,56	*0,15*	**S***
**26**	747	7	10.51 (R)	*0,6*	0.08 (R)	*0,02*	**T**
**27.1**	692	2	15.06 (R)	*0,6*	-0.10 (R)	*0,02*	**?**
**27.2**	683	15	-2,81	*1,83*	-0,68	*0,06*	**S**
**28**	610	12	16,95	*2,7*	0,65	*0,1*	**S**
**29**	588	2	N/A	N/A	N/A	N/A	**N/A**
**30**	576	22	6,05	*1,15*	0,71	*0,04*	**S**
**31**	541	18	7,53	*3,46*	0,97	*0,12*	**S**
**32**	491	12	11.63 (R)	*0,6*	0.10 (R)	*0,02*	**?**
**33**	434	9	9	*3,23*	0,54	*0,12*	**S**
**34**	305	10	12,21	*3,34*	0,3	*0,12*	**S**
**35**	235	17	15,33	*4,77*	0,54	*0,17*	**S**
**36**	216	6	10,87	*3,11*	0,42	*0,11*	**S**
**37**	205	10	0.64 (R)	*0,6*	0.07 (R)	*0,02*	**T**
**38**	171	8	-8,49	*3,72*	-0,3	*0,13*	**S**
**39**	125	6	1,11	*3,72*	0,36	*0,13*	**S***
**40**	-7	8	16,51	*3,01*	0,46	*0,11*	**S***
**41**	-42	8	13.3 (R)	*0,6*	0.06 (R)	*0,02*	**T***
**42**	-81	11	14,84	*4,44*	0,35	*0,16*	**S**
**43**	-103	9	8,06	*3,85*	0,44	*0,14*	**S***
**44**	-129	20	14,26	*3,01*	1,94	*0,11*	**S**
**45**	-174	7	13,53	*4,14*	1,16	*0,15*	**S**
**46.1**	-212	30	10,02	*1*	0,56	*0,03*	**S**
**46.2**	-230	19	17,94	*5,3*	0,48	*0,19*	**S**
**46.3**	-250	20	6,9	*2,52*	-0,53	*0,09*	**S**
**47**	-335	5	14.28 (R)	*0,6*	-0.09 (R)	*0,02*	**?**
**48**	-348	7	9.58 (R)	*0,6*	0.02 (R)	*0,02*	**T***
**49**	-429	40	11,39	*0,93*	0,97	*0,03*	**S**
**50.1**	-469	19	16.45 (R)	*0,6*	0.01 (R)	*0,02*	**T***
**50.2**	-476	7	13.01 (R)	*0,6*	-0.01 (R)	*0,02*	**T***
**50.3**	-484	11	11.1 (R)	*0,6*	0.08 (R)	*0,02*	**T***
**51**	-531	15	9.25 (R)	*0,6*	0.09 (R)	*0,02*	**?**

Supplementary Table 1 – Listing of identified volcanic events. Dating is provided by Sigl *et al.*[1] Blue and red dates indicate events identified as southern hemisphere volcanic eruptions and tropical eruptions in Sigl *et al.*[1], respectively. Grey dates are not mentioned in Sigl *et al.*[1] The flux is the volcanic sulfate deposition flux, corrected from background, calculated from concentrations measured in this study. **Δ^{33}S** is the maximum **^{33}S-excess** (in absolute value) observed on the event, it has been corrected from background, unless otherwise indicated (by (R), standing for "Raw value"). The fifth column is the volcanic source (Stratospheric/Tropospheric) inferred through the isotopic result. N/A, "?" stand for non-analyzed and uncertain source (ambiguous isotopic signal) respectively. Sources marked with an asterisk (*) are discordant with Sigl *et al.*[1] index.

Event	Date / year (based on Sigl et al. 2015)	Flux / kg.km-2	Δ^{17}O / ‰
**10**	**1642**	16	3,10
**13**	**1458**	42	3,22
**15**	**1346**	15	2,3
**16.4 Samalas**	**1259**	85	0,76
**17**	**1230**	30	2,56
**22**	**960**	13	4,69
**30**	**576**	22	1,22
**31**	**541**	18	3,90
**35**	**235**	17	4,13
**37**	**205**	10	3,57
**38**	**171**	8	2,61
**44**	**-129**	20	3,75
**46.1**	**-212**	30	3,06
**49**	**-429**	40	0,45

Supplementary Table 2: ^{17}O-excess measured in sulfate from 14 stratospheric events. Data are not corrected from background, and are given with a 1σ-uncertaintiy of 0.3 ‰ [2].

Event	δ^{33}S (‰)	δ^{34}S (‰)	δ^{36}S (‰)	$\Delta^{33}S$ (‰)	$\Delta^{36}S$ (‰)	$[SO_4^{2-}]$ (ppb)
**1**						62
**2**						83
**3**						72
**4**						60
**5**						67
**6**	7.89	15.24	29.81	0.07	0.65	66
**7**						80
**8**						65
**9**						71
**10**	6.42	12.68	24.53	-0.09	0.30	63
**11**						80
**12**						86
**13**	7.28	14.04	27.53	0.07	0.69	90
**14**						84
**15**	8.50	16.57	32.39	0.00	0.68	77
**16**	8.34	16.09	31.15	0.09	0.36	79
**17**	7.83	15.15	26.81	0.06	-2.17	67
**18**						63
**19**						76
**20**						89
**21**						91
**22**	7.56	14.53	28.76	0.10	0.97	90
**23**						83
**24**						86
**25**						86
**26**						84
**27**						82
**28**						75
**29**						83
**30**	7.40	14.26	27.84	0.08	0.57	101
**31**	7.84	15.15	29.48	0.07	0.49	103
**32**						81
**33**						88
**34**						79
**35**	7.63	14.76	28.79	0.05	0.57	81
**36**						80
**37**	7.06	13.63	26.80	0.07	0.75	97
**38**	7.15	13.91	27.32	0.01	0.72	103
**39**						90
**40**						105
**41**						88
**42**						98
**43**						97
**44**	6.38	12.31	23.71	0.06	0.18	81
**45**						88
**46**	8.12	15.70	30.49	0.07	0.46	63
**47**						91
**48**	7.86	15.26	29.63	0.03	0.45	97
**49**	8.32	15.93	30.53	0.14	0.04	90
**50**	8.69	16.81	32.36	0.07	0.18	78
**51**	8.95	17.37	33.54	0.05	0.28	86
**Average**	7.73	14.97	28.97	0.05	0.34	82
**St. Dev (1σ)**	0.71	1.36	2.63	0.05	0.67	12
**2σ**	1.4	2.7	5.3	0.1	1.3	23

Supplementary Table 3: Background composition of volcanic events, and background average values, used to correct volcanic data when isotopic background values were not known. To estimate the volcanic fraction in each sample, the background sulfate concentration specific to this event, as presented in this table, was used. Note that one event can gather several volcanic peaks (for example event 6 includes Tambora and UE 1809 eruptions) close to each other, which makes difficult the sampling of separate background. A unique background value is considered for these eruptions in such cases.

Volcanic fraction	1σ - $\delta^{34}S$	1σ - $\Delta^{33}S$	1σ - $\Delta^{36}S$
**0.9**	0.68	0.02	0.24
**0.8**	0.82	0.03	0.30
**0.7**	1.03	0.04	0.41
**0.6**	1.34	0.05	0.55
**0.5**	1.80	0.06	0.78
**0.4**	2.53	0.09	1.13
**0.3**	3.75	0.13	1.69
**0.2**	6.21	0.23	2.87
**0.1**	13.70	0.52	6.49

Supplementary Table 4: Uncertainties arising when isotopic values are corrected from the background contribution, obtained through Monte Carlo error propagation method.

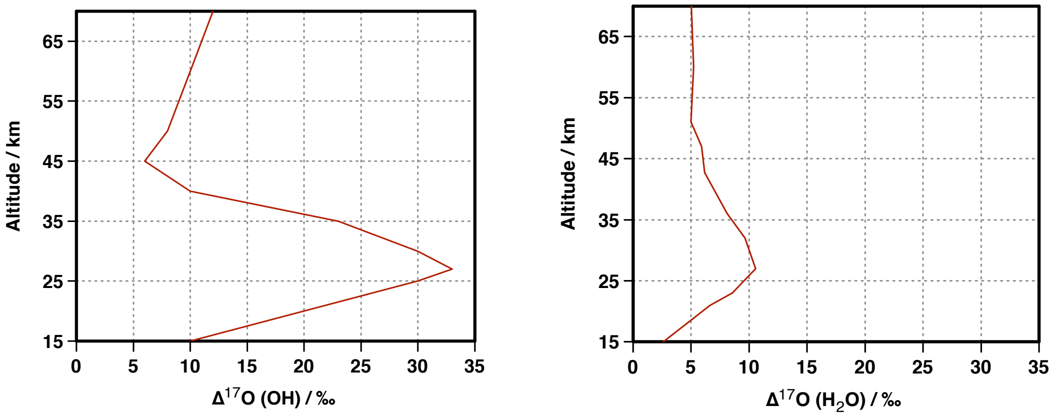

Supplementary Figure 1 – Left: Schematic representation of the evolution of $\Delta^{17}O$ (OH) with altitude in the stratosphere, adapted from Zahn *et al.*[3] OH 17-excess is maximum around 30 km of altitude. It then decreases by half above 40 km. In that sense, the ^{17}O-excess of sulfate could reflect the altitude of formation of the aerosols in the stratosphere.

Right: A similar trend is followed by H_2O with a lower magnitude (adapted from Zahn *et al.*[3] fig. 10).

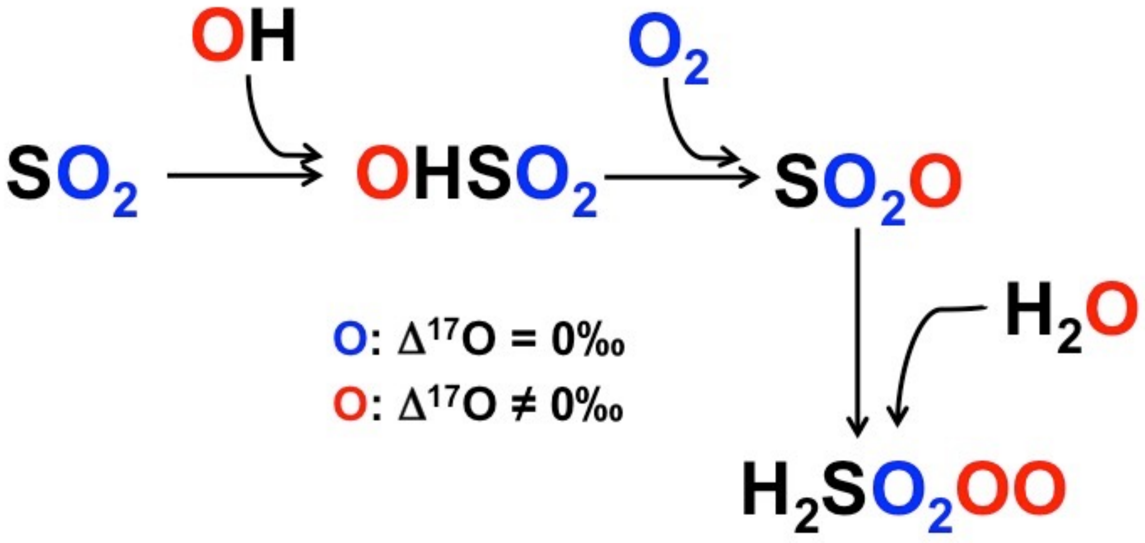

Supplementary Figure 2 – Mechanisms involved in SO_2 oxidation through reaction with OH radicals in the stratosphere highlighting steps involved in the transfer of a ^{17}O-excess to the end product sulfate molecule. Note that ^{17}O-excess has different value in OH and H_2O.

Supplementary References

1 Sigl, M. *et al.* Timing and climate forcing of volcanic eruptions for the past 2,500 years. *Nature* **523**, 543-549, doi:10.1038/nature14565 (2015).

2 Ishino, S. *et al.* Seasonal variations of triple oxygen isotopic compositions of atmospheric sulfate, nitrate, and ozone at Dumont d'Urville, coastal Antarctica. *Atmospheric Chemistry and Physics*, 3713-3727, doi:10.5194/acp-17-3713-2017 (2017).

3 Zahn, A., Franz, P., Bechtel, C., Grooß, J.-U. & Röckmann, T. Modelling the budget of middle atmospheric water vapour isotopes. *Atmospheric Chemistry and Physics* **6**, 2073-2090 (2006).
